# Association of Serum Albumin, Globulin, and Transferrin Levels in Children of Poorly Managed Celiac Disease

**DOI:** 10.1155/2023/5081303

**Published:** 2023-02-01

**Authors:** Komal Siddiqui, Arsalan Ahmed Uqaili, Salma Memon, Tazeen Shah, Saima Naz Shaikh, Ali Raza Memon

**Affiliations:** ^1^Institute of Biotechnology and Genetic Engineering, University of Sindh, Jamshoro, Pakistan; ^2^Department of Physiology, Liaquat University of Medical and Health Sciences, Jamshoro, Pakistan; ^3^Department of Biochemistry, Liaquat University of Medical and Health Sciences, Jamshoro, Pakistan

## Abstract

**Background:**

Celiac disease (CD) is an autoimmune genetic disorder in which gluten protein causes inflammation of the intestinal enterocytes. CD diagnosis in most cases is delayed or mistreated due to its varied clinical features. We aimed to evaluate the protein profile imbalance in different CD groups of children, which could help aid in the diagnosis and proper management of the disease. *Methodology*. This was a cross-sectional study with a nonrandom purposive sampling technique. All samples were taken from tertiary care hospitals of Hyderabad, Pakistan. In total, there were 175 children (age 3-15 years) divided into five equal groups (*n* = 35), namely, group A (control), group B (celiac diagnosed), group C (celiac-like symptoms), group D (celiac with type 1 diabetes mellitus), and group E (type 1 diabetes mellitus only). Clinical symptoms and laboratory parameters were analyzed among all the groups. Sera proteins, albumin, globulins, and transferrin levels were evaluated and compared with healthy individuals.

**Results:**

The albumin in serum of celiac groups B and C was 3.0 g/dl and 2.8 g/dl, respectively. While in diabetic patients with CD, it is 2.7 g/dl. The globulin levels were raised among all the celiac groups with typical GIT symptoms. The highest transferrin was observed in group B, celiac patients with severe anemia. Patients were not on GFD, hence had no or less recovery and had chronic symptoms of celiac.

**Conclusion:**

The misdiagnosis and poor management of celiac leads to chronic villous atrophy with imbalance in metabolic profile. Serum analysis of albumin, globulins, and transferrin may help in the diagnosis and proper management of the disease to recover the celiac symptoms.

## 1. Introduction

Celiac enteropathy is a common food-related disorder with a prevalence of 1-2% worldwide [1]. Moreover, it is overpresented in type 1 diabetes mellitus. Celiac disease (CD) is a lifelong autoimmune condition in which genetically susceptible individuals are allergic to dietary gluten. Gluten is a major protein found in wheat, barley, and rye grains [1]. Gluten causes inflammation of the small intestine by eliciting T and B cell-mediated immune responses, which cause damage to enterocytes in the lining of the intestinal mucosa. Continuous exposure to gluten results in villous atrophy and crypt hyperplasia. The only treatment for CD is to completely restrict gluten consumption [2]. In the early days, it was thought to be a childhood malabsorption disorder, but now, it is well established that CD could be diagnosed at any age having varied clinical manifestations [3]. The major genetic risk factors of CD are HLA-DQ2 and HLA-DQ8 heterodimers present on the surface of antigen-presenting cells [4, 5]. Modern diagnostic methods now emphasize on genetic profiling for the known celiac alleles HLA-DQA1∗05, HLA-DQB1∗02, and HLA-DQB1∗03:02 to rule out misdiagnosis or related GIT problems [6].

In many cases, despite the availability of advanced diagnostic approaches, CD remains undiagnosed. One major reason behind late diagnosis is the varied clinical features of disease. Some people have mild to severe symptoms, while some are asymptomatic. In poorly managed CD, a lot of essential nutrients are not absorbed properly from the atrophied intestine, leading to many complications. Most prominent ones are diarrhea, growth retardation, and fatigue, while in chronic conditions, anemia, hyposplenism, osteoporosis, hypoalbuminemia, arthritis, neoplasm, and other metabolic imbalances have been known to occur [7, 8].

Due to flattening of mucosa, a lot of protein is lost from the lesioned intestine along with malabsorption of many macro- and micronutrients. Chronic stimulation of the immune system as in mismanaged celiac cases often results in hypoalbuminemia and increased levels of globulins. Albumin and globulin are the major blood serum proteins. The difference in total protein and albumin is frequently used as a screening tool for the detection of various autoimmune inflammatory diseases or in latent infections [9–12]. One important *β*-globulin synthesized in the liver is transferrin protein. The main role of transferrin is the transportation and maintenance of iron in the serum [13]. Untreated CD cases suffer from iron deficiency anemia (IDA) which results in higher peaks of transferrin, reflecting upsurge in protein translation [14].

Our study was aimed at evaluating albumin, globulin, and transferrin imbalance in children who have been recently diagnosed with CD and CD in comorbidity with type 1 diabetes mellitus. We determined whether metabolic profile in poorly managed cases is necessary to be assessed during the diagnosis of these patients. To our knowledge, negligible data is available about CD scenario in our country. This survey is an initiative towards the status of celiac in Hyderabad, Pakistan.

## 2. Materials and Methods

This was a cross-sectional study with nonrandom purposive sampling technique, comprising of 175 pediatric patients with typical celiac symptoms. There were 112 males and 63 females with mean age 8.7 ± SD 1.2 years. The samples were collected from the three tertiary care hospitals in the locality of Hyderabad, Pakistan, namely, Liaquat University of Medical and Health Sciences (LUMHS) hospital, Isra University hospital, and Asian Institute of Medical Sciences (AIMS) hospital. The individuals were divided into 5 groups (*n* = 35): control (group A), diagnosed cases of celiac disease (group B), patients with celiac-like symptoms (group C), type 1 diabetes patients with celiac-like symptoms (group D), and type 1 diabetes patients (group E). A detailed questionnaire was initially developed with the help of the Canadian Celiac Society to know the status of the disease in each patient.

### 2.1. Albumin and Globulin Test

Albumin and globulin measurements were performed using the automated analyzer cobas® 6000 (Roche Diagnostics). The normal concentration of albumin and globulin in the blood is 3.5-5.5 g/dl and 2.0-3.5 g/dl, respectively [15].

### 2.2. Transferrin Concentration

Total serum transferrin concentration was determined by using human transferrin receptor kit (by Bioassay Technology Laboratory) and analyzed with ELISA reader (DIASource 2000).

### 2.3. Data Collection and Analysis

The data of controls and patients were gathered by the help of a questionnaire. The data focused on the patients already tested for anti-tTG antibodies (group B). The patients having celiac-like symptoms were tested for anti-tTG (groups C and D). Statistical analysis and data interpretation were carried out by using SPSS version 21.0, and significance was calculated using ANOVA and post hoc Tukey's analysis taking statistical significance with *p* value ≤ 0.05.

## 3. Results

### 3.1. Symptoms among Celiac Groups

Among groups B, C, and D, the patients suffered from typical CD symptoms like anemia, weight loss, and diarrhea.  Table 1 summarizes the signs seen in the distinct groups. Some of the respondents showed atypical symptoms as well like constipation, mouth ulcer, and mood swings.

### 3.2. Serum Albumin, Globulin, and Transferrin Levels

The normal levels of albumin in serum should be 3.5-5.5 g/dl (256-261). In celiac group B and group C, the values were 3.0 g/dl and 2.8 g/dl, respectively, while in diabetic patients with CD, the mean was observed to be 2.7 g/dl. As in inflammatory disorders, total globulin levels are raised. The highest peaks were observed in groups B and D, followed by group C (Table 2). For healthy individuals, the range of transferrin should be 170-370 mg/dl, which was observed in group A. For group B, the highest transferrin was observed, because the majority of patients had severe anemia and were not on a strict gluten-free diet due to symptoms of celiac were persistent with less recovery. Moreover, for group C, the ranges were observed to be toward the higher end. In participants suffering from both diabetes and celiac (group D), the transferrin was observed to be 406.4 mg/dl (Table 2). Furthermore, post hoc analysis was conducted by comparing albumin (Table 3), globulin (Table 4), and transferrin (Table 5) levels individually with the healthy controls.

## 4. Discussion

Celiac disease is a common food-related disorder around the world having a prevalence of 1-2% with 0.6% prevalence reported in Asia [16]. Though in Pakistan, it seems to be a common autoimmune disease, often, it is mistaken as common diarrhea and gastroenteritis. Based on clinical manifestations, CD can be classical, silent, or latent. Some of the nonclassical complications include osteoporosis, abdominal pain, iron deficiency anemia, amenorrhea, and constipation [17]. Other less common silent clinical features are protein loss, neurological problems, ESR, increased imbalance in liver enzymes, and reproductive anomalies [18–21]. In our study, we selected patients who were already diagnosed with CD and analyzed their symptoms. Along with that, children with CD like symptoms (group C) and children with DM1-CD (group D) were also observed. In the celiac groups, children showed hypoalbuminemia with the lowest level (2.7 g/dl) in group D. Albumin deficiencies among celiac patients have been described by many authors [22, 23]. Newton and Singer in 2012 reported many metabolic deficiencies like iron, folates, and lower albumin levels in celiac patients and Tumay et al. studied various parameters among different groups of children and observed lower levels of albumin, cholesterol, calcium, and hemoglobin [24]. A study in Turkey showed hypoalbuminemia among 9.5% of celiac individuals [25]. It has been observed that here in Pakistan, the follow-up for the disease is not frequent, due to which many complications become chronic. Also, the delays in diagnosis and poor awareness of the gluten-free diet complicate the exact diagnosis. Another frequent laboratory findings of celiac disease patients under gluten-containing diet could be transaminase levels; the hypertransaminasaemia and serum actin Ig-A antibodies are being used in some parts of the world frequently but not all over as standard practices [26].

Increased globulin level indicates that the body is under some malfunctioning. Since autoimmune disorders cause inflammatory reactions, the highest globulin peak (4.6 g/dl) was also observed in group D, which was DM1 with CD. The normal range in healthy folks should be 2-3.5 g/dl. Since albumin and globulins play an essential role in immunity and inflammation processes, we speculate that an elevation in globulins could be a helpful marker for detecting silent/subclinical celiac disease. These not only depict the nutritional status but also may help in knowing hidden chronic inflammatory disorders [27, 28].

Transferrin (beta globulin) has been observed to be very high in CD children (groups B, C, and D), while there is a reciprocal decrease in transferrin iron saturation and corresponding increase in TIBC [29, 30]. In this study, the normal range of transferrin 170-370 mg/dl was observed in group A (Table 2). For group B, the highest transferrin was observed. Moreover, for group C, the ranges were observed to be toward the higher end. In participants suffering from both diabetes and celiac (group D), the transferrin was observed to be 406.4 mg/dl. The results are consistent with a study, in which almost 50% of the cases had higher transferrin concentrations and TR index, especially in nutritionally deficient celiac adults and children. Growth was slower in females compared to male participants when compared to controls [31]. Moreover, Tursi et al. observed iron deficiency among one-third of the celiac patients, and due to iron depletion, an elevation in TfR-R index was found. In inflammatory condition, testing for both transferrin receptor and ferritin is a more reliable method to identify related iron deficiencies [32, 33].

## 5. Conclusion

Celiac patients suffer from a varied class of symptoms, and it is still very hard to draw any association between unusual features with silent/subclinical CD. Thus, in our study, we suggest that a complete metabolic profile should be carried out for patients with severe malnutrition. Moreover, if there are mild symptoms of CD with imbalance in albumin and globulin count, then serological testing for anti-tTG is advised for accurate diagnosis and for proper management of CD.

## Figures and Tables

**Table 1:** Common symptoms being suffered by patients.

	Anemia	Weight loss	Diarrhea	Abdominal pain	Nausea	Constipation	Bruising of skin	Muscle cramps	Swelling in peripheries	Growth retardation	Eczema	Mouth ulcers	Others
Group B	29	31	28	26	8	6	21	9	13	19	18	7	7
Group C	26	23	21	29	6	4	16	11	10	21	19	3	4
Group D	21	11	17	14	10	7	19	7	19	28	11	1	6
Group E	18	10	3	9	12	3	4	2	15	7	4	00	5

**Table 2:** Serum protein analysis of celiac patients.

	Group A	Group B	Group C	Group D	Group E	*p* value
Albumin (3.5 to 5.5 g/dl)	4.4 ± 0.6	3.0 ± 0.5	2.8 ± 0.4	2.7 ± 0.4	3.1 ± 0.6	0.0001
Globulin (2.0 to 3.5 g/dl)	2.8 ± 0.4	4.7 ± 0.4	4.0 ± 0.6	4.6 ± 0.3	3.8 ± 0.5	0.0001
Transferrin (170 to 370 mg/dl)	271.5 ± 39.1	411.5 ± 21.5	387.7 ± 22.4	406.4 ± 21.4	368.8 ± 22.0	0.001

*p* value calculated by ANOVA.

**Table 3:** Post hoc analysis of serum albumin.

		Mean difference	*p* value
Group A	Group B	1.4	0.0001
	Group C	1.6	0.0001
	Group D	1.7	0.0001
	Group E	1.3	0.002

Group B	Group C	0.2	0.061^∗^
	Group D	0.3	0.053^∗^
	Group E	-0.1	0.069^∗^

^∗^ *p* value is >0.05.

**Table 4:** Post hoc analysis of serum globulin.

		Mean difference	*p* value
Group A	Group B	-1.9	0.001
	Group C	-1.2	0.001
	Group D	-1.8	0.001
	Group E	-1.0	0.001

Group B	Group C	0.7	0.051
	Group D	0.1	0.073^∗^
	Group E	0.9	0.001

^∗^ *p* value is >0.05.

**Table 5:** Post hoc analysis of serum transferrin.

		Mean difference	*p* value
Group A	Group B	-140	0.0001
	Group C	-116.2	0.0001
	Group D	134.9	0.0001
	Group E	-97.3	0.001

Group B	Group C	23.8	0.01
	Group D	5.4	0.08^∗^
	Group E	42.7	0.01

^∗^ *p* value is >0.05.

## Data Availability

The results and data used to support the findings of this study were collected in the process of doctoral thesis of Dr Komal Siddiqui. The data is still under the process of analysis through gene sequencing analysis so due to that reason, the data cannot be made freely available. Requests for access to these data should be made to the corresponding author Dr Arsalan Ahmed Uqaili (Assistant Professor, Department of Physiology, LUMHS, Pakistan, +923202755701, arsalanuqaili@gmail.com) and Dr Komal Siddiqui (Assistant Professor, University of Sindh, Pakistan, siddiqui.komal@gmail.com). 2 articles are already published which are freely available for citation at given links; any dataset request will be promptly entertained (https://www.ncbi.nlm.nih.gov/pmc/articles/PMC7982179/ and https://www.kmuj.kmu.edu.pk/article/view/20553).
